# Single-Cell Transcriptional Changes in Hypothalamic Corticotropin-Releasing Factor–Expressing Neurons After Early-Life Adversity Inform Enduring Alterations in Vulnerabilities to Stress

**DOI:** 10.1016/j.bpsgos.2021.12.006

**Published:** 2021-12-23

**Authors:** Annabel K. Short, Christina W. Thai, Yuncai Chen, Noriko Kamei, Aidan L. Pham, Matthew T. Birnie, Jessica L. Bolton, Ali Mortazavi, Tallie Z. Baram

**Affiliations:** a Department of Anatomy and Neurobiology, University of California Irvine, Irvine, California; b Department of Pediatrics, University of California Irvine, Irvine, California; c Department of Developmental and Cell Biology, University of California Irvine, Irvine, California; d Department of Neurology, University of California Irvine, Irvine, California

**Keywords:** CRF, Early-life adversity, Epigenomics, Hypothalamus, Mental illness, Single-cell transcriptomics, Stress

## Abstract

**Background:**

Mental health and vulnerabilities to neuropsychiatric disorders involve the interplay of genes and environment, particularly during sensitive developmental periods. Early-life adversity (ELA) and stress promote vulnerabilities to stress-related affective disorders, yet it is unknown how transient ELA dictates lifelong neuroendocrine and behavioral reactions to stress. The population of hypothalamic corticotropin-releasing factor (CRF)–expressing neurons that regulate stress responses is a promising candidate to mediate the long-lasting influences of ELA on stress-related behavioral and hormonal responses via enduring transcriptional and epigenetic mechanisms.

**Methods:**

Capitalizing on a well-characterized model of ELA, we examined ELA-induced changes in gene expression profiles of CRF-expressing neurons in the hypothalamic paraventricular nucleus of developing male mice. We used single-cell RNA sequencing on isolated CRF-expressing neurons. We determined the enduring functional consequences of transcriptional changes on stress reactivity in adult ELA mice, including hormonal responses to acute stress, adrenal weights as a measure of chronic stress, and behaviors in the looming shadow threat task.

**Results:**

Single-cell transcriptomics identified distinct and novel CRF-expressing neuronal populations, characterized by both their gene expression repertoire and their neurotransmitter profiles. ELA-provoked expression changes were selective to specific subpopulations and affected genes involved in neuronal differentiation, synapse formation, energy metabolism, and cellular responses to stress and injury. Importantly, these expression changes were impactful, apparent from adrenal hypertrophy and augmented behavioral responses to stress in adulthood.

**Conclusions:**

We uncover a novel repertoire of stress-regulating CRF cell types differentially affected by ELA and resulting in augmented stress vulnerability, with relevance to the origins of stress-related affective disorders.

Mental health and vulnerability to neuropsychiatric disorders involve the interplay of genes and environment during sensitive developmental periods (1, 2, 3). Genetic and environmental factors contribute to the development and maturation of neurons, synapses, and brain circuits, which in turn drive long-lasting phenotypes.

Early-life adversity (ELA) promotes vulnerability to stress and stress-related affective disorders, but the mechanisms for these enduring phenotypes are incompletely understood (1,2,4,5). Stress-sensitive corticotropin-releasing factor (CRF)–expressing neurons residing in the hypothalamus are candidate mediators of the long-lasting effects of ELA because they contribute to both hormonal and behavioral response to stress (6, 7, 8, 9, 10). CRF release from hypothalamic paraventricular nucleus (PVN) neurons induces pituitary adrenocorticotropic hormone secretion, which stimulates corticosterone release from the adrenals (11, 12, 13). CRF may also signal to local CRF-responsive neurons (14,15).

CRF-expressing PVN neurons have traditionally been divided into three major subpopulations (16, 17, 18, 19). Preautonomic neurons project to the brainstem and spinal cord (20); magnocellular neurons coexpressing arginine vasopressin or oxytocin project to the posterior pituitary (21); and neuroendocrine parvocellular cells, the predominant population of CRF cells within the PVN (22), project to the median eminence and release CRF (16). In contrast to this traditional classification, recent single-cell analyses of hypothalamic transcriptomes have identified multiple molecular-defined clusters of CRF-expressing neurons that may represent different subpopulations or distinct neuronal functional states (23, 24, 25, 26).

CRF expression and the connectivity of CRF cells in PVN are modulated by early-life experiences. Optimal rearing conditions, including augmented maternal care, repress CRF expression (27, 28, 29), whereas adversity in early life may increase (29,30) or decrease (31) the peptide’s expression. Augmented maternal signals reduce excitatory synapses to CRF cells (27,32), whereas adversity increases excitatory glutamatergic transmission to the same cell population (30,33). There is evidence that changes in synaptic neurotransmission induce transcriptional reprogramming of neurons (34, 35, 36). These enduring transcriptomic alterations in CRF cells might underlie the augmented stress responses induced by ELA (37, 38, 39, 40). However, it remains unclear how ELA-influenced gene expression programs CRF cells, if such changes are specific to subpopulations, and whether the transcriptional changes are associated with increased vulnerabilities to stress throughout life.

Here, we used a model of ELA that provokes major alterations in cognitive and emotional outcomes (2,41, 42, 43, 44, 45, 46, 47, 48, 49, 50, 51, 52, 53, 54, 55, 56), including augmented responses to stress (31,33,57). We focused on the change in gene expression profiles of stress-regulating PVN CRF neurons following ELA in male mice. We used single-cell RNA sequencing to probe the effects of ELA on gene expression programs in distinct neuronal populations, determine the potential selectivity of the effects of ELA, and identify their downstream consequences.

## Methods and Materials

### Animals

*Crh*-IRES-Cre^+/+^ (58) dams were paired with Ai14 tdTomato (59) males, both on a C57BL6 background. The resulting offspring were *Crh*-IRES-Cre;Ai14 tdTomato, which express tdTomato with nearly full overlap of native CRF (22,60). Mice were housed in a 12-hour light/dark cycle with ad libitum food and water. All experiments were carried out in accordance with the Institutional Animal Care and Use Committee at the University of California, Irvine, and were consistent with federal guidelines.

### ELA Paradigm Cages With Limited Bedding and Nesting

**Figure 1:**
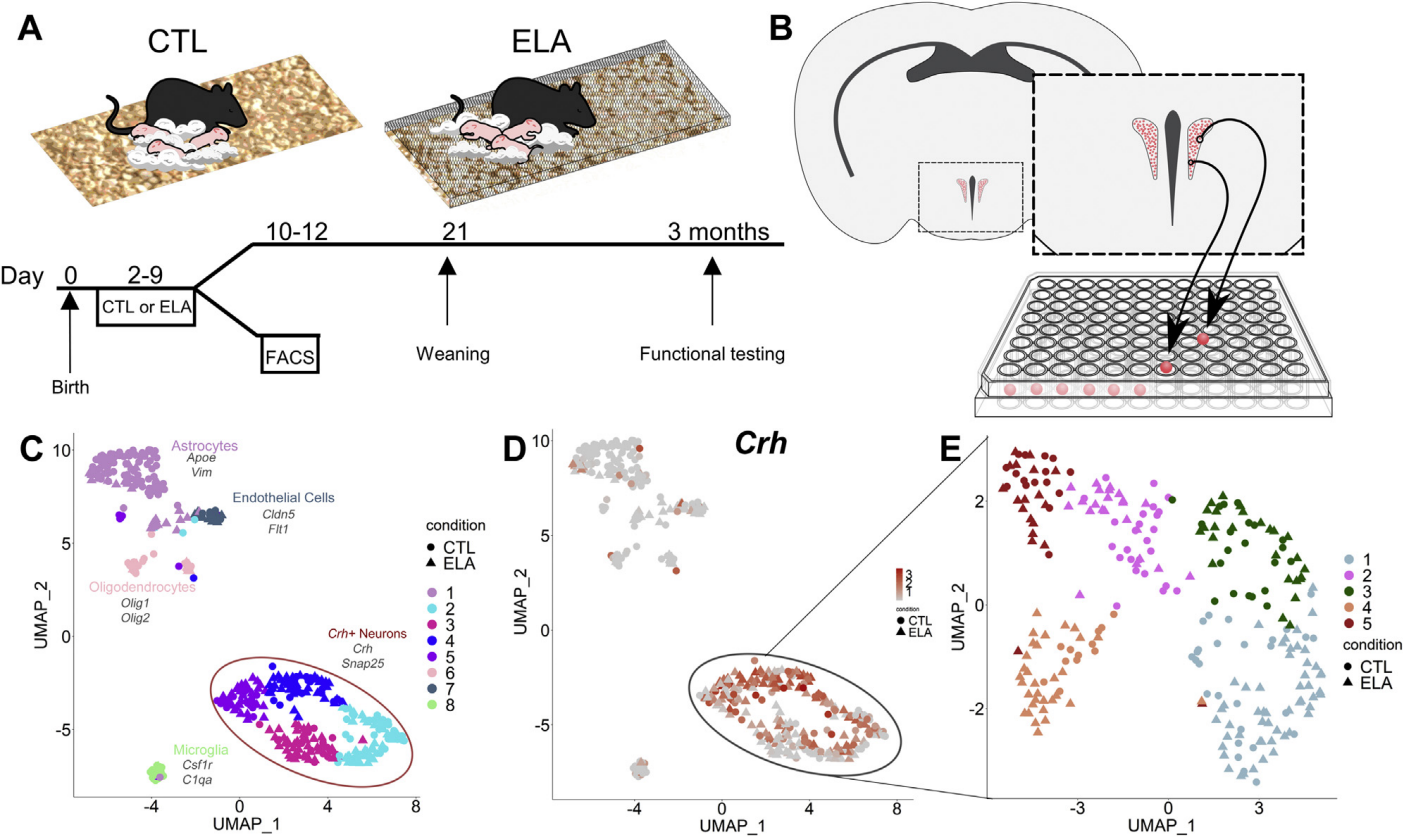
Single-cell RNA sequencing of corticotropin-releasing factor cells in the hypothalamic paraventricular nucleus reveals distinct neuronal populations. **(A)** Mice exposed to either CTL or ELA conditions during the 10 days of life were either kept for functional testing or used for collection of paraventricular nucleus cells during postnatal days 10–12. **(B)** Corticotropin-releasing factor cells were collected from *Crh*-IRES-Cre;Ai14 tdTomato mice by dissecting a paraventricular nucleus–containing tissue block, sorted into individual wells using FACS, and sequenced. **(C)** UMAP was performed on cluster-sorted cells and revealed eight distinct groups of cells, the majority of which were classified as neurons based on expression of *Snap25*. **(D)** These neurons, the majority of *Crh*-expressing cells, further clustered into five subpopulations **(E)**. CTL, control; ELA, early-life adversity; FACS, fluorescence-activated cell sorting; UMAP, Uniform Manifold Approximation and Projection.

We imposed ELA on neonatal mice, simulating poverty by limiting nesting and bedding materials in cages during the early developmental period as described previously and in Supplemental Methods (Figure 1A) (31,41,47). For RNA sequencing, pups remained on the limited bedding and nesting paradigm until tissue was collected on postnatal day (P) 10 to P12. For experiments in adulthood, experimental groups were transferred to standard cages on P10 and were weaned on P21; mice were housed by sex with littermates. All mice used in this study were male mice, and females were the focus of further studies.

### Single-Cell Preparation

Male P10 to P12 pups were killed via decapitation, brains were removed immediately and placed on ice, and the PVN was dissected (Figure 1B and Supplemental Methods) (control: *n* = 50 pups from 14 litters, and ELA: *n* = 38 pups from 10 litters). The trimmed slices were placed into papain (20 units/mL) and homogenized. Supernatant was removed, and cells were resuspended in 500 μL 2% fetal bovine serum in phosphate-buffered saline. Immediately before sort, cell suspension was run through a 70-μM filter and washed with 500-μL 2% fetal bovine serum in phosphate-buffered saline. Cells were sorted into 8 well strip tubes and immediately spun down at 4 °C and frozen on dry ice (see Supplemental Methods for full details and Figure 1B).

### RNA Sequencing Pipeline

Tdtomato-positive hypothalamic cells from both ELA (153 cells) and control (101 cells) mice were processed using the SmartSeq2 RNA sequencing protocol and Illumina Library prep (Nextera XT DNA Library Preparation Kit) and sequenced using Illumina NextSeq500 sequencer (Illumina) to an average depth of 3.8 million reads per cell. Reads were mapped and quantified using kallisto (61). Cells were filtered for >1000 expressed genes per cell, and genes expressed in four or more cells were included. The resulting transcript per million matrix was quantile normalized and clustered using the R package Seurat followed by nonlinear dimensional reduction (62). ComplexHeatmap was used for heatmap generation (63). Metascape (64) was used to determine Gene Ontology and pathways.

### Immunofluorescence Staining

Perfused brains were sectioned coronally (20 μm; 1:6 series) Immunofluorescence was performed on brain sections derived from P10 male tdTomato-Crh (*Crh-*IRES*-*Cre;Ai14) transgenic mice as described (60,65) and imaged using confocal microscopy (see Supplemental Methods for details).

### Tests of CRF+ PVN Cell Function

#### Looming Shadow Task

The looming shadow task is associated with PVN CRF neuronal activity (8). The entire task was performed in the dark active phase as described previously (66) and in the Supplement. Response to the looming stimulus was scored as absent, freezing, or escape behavior and recorded both live and on video (control: *n* = 15 mice from 4 litters; ELA: *n* = 15 mice from 5 litters). Percentage escape was calculated as (number of escapes/number of total trials) × 100.

#### Stress in Adulthood

Acute (1 hour) concurrent stresses (67,68) were imposed, including restraint, bright light, and loud music (67, 68, 69, 70, 71) (see Supplemental Methods for details).

#### Corticosterone Assay

Serum corticosterone was analyzed using the corticosterone EIA Kit (Cayman) according to the manufacturer’s instructions (control baseline: *n* = 5 mice from 1 litter, control stress: *n* = 10 mice from 3 litters; ELA baseline: *n* = 5 mice from 2 litters, ELA stress: *n* = 7 mice from 2 litters). For full methods, see Supplemental Methods.

#### Adrenal Gland Collection

In a separate cohort of mice (control: *n* = 6 mice from 2 litters; ELA: *n* = 7 mice from 3 litters), gross dissection isolated both adrenals, which were weighed together. Adrenal size is expressed per body weight (see Supplemental Methods for details).

### Statistical Considerations and Analyses

Where possible, data collection and analyses were performed blinded to treatment group. Statistical analyses for χ^2^ tests and functional tests were performed using GraphPad Prism 9.0 (GraphPad Software) using a Student *t* test with significance set at *p* ≤ .05. All other analyses were performed using R studio version 4.0.2 (72). Graphs show the mean ± standard error of the mean.

## Results

### CRF-Expressing Neurons in the Developing Mouse Hypothalamus Belong to Distinct Populations With Unique Gene Expression Profiles

The hypothalamic PVN harbors several types of CRF-expressing neurons, including neuroendocrine cells releasing CRF into the portal bloodstream and others projecting to specific brain areas (16,17). The majority of PVN CRF-expressing (CRF+) cells coexpress glutamate, a key excitatory neurotransmitter (73,74), whereas others express genes associated with inhibitory GABAergic (gamma-aminobutyric acidergic) neurotransmission (75). To delineate these populations in developing mouse PVN and determine their potential contribution to the phenotypic consequences of ELA, we used single-cell transcriptomics.

Whereas weight gain was slightly slower in ELA mice (Figure S2A), harvested cells did not cluster based on age or weight of the mice or harvest batch (Figure S1A–C). Individual cells passing filter criteria were characterized as CRF+ neurons or microglia, astrocytes, or endothelial cells, based on marker genes (Figure 1C), and the latter were excluded from further analyses. We also excluded cells expressing *Pgr15l*, which is coexpressed with CRF in GABAergic neurons residing at the border of the dorsomedial hypothalamus and PVN (Figure S1D) (24,76). Focusing on cells coexpressing *Crh* and the neuronal synaptic protein encoding gene *Snap25* (Figure 1D), we characterized them using shared nearest neighbor clustering (using the Seurat package) (62), which segregated CRF+ neurons into five distinct clusters (Figure 1E).

### ELA Significantly Affects Gene Expression Programs in Hypothalamic CRF Cells

**Figure 2:**
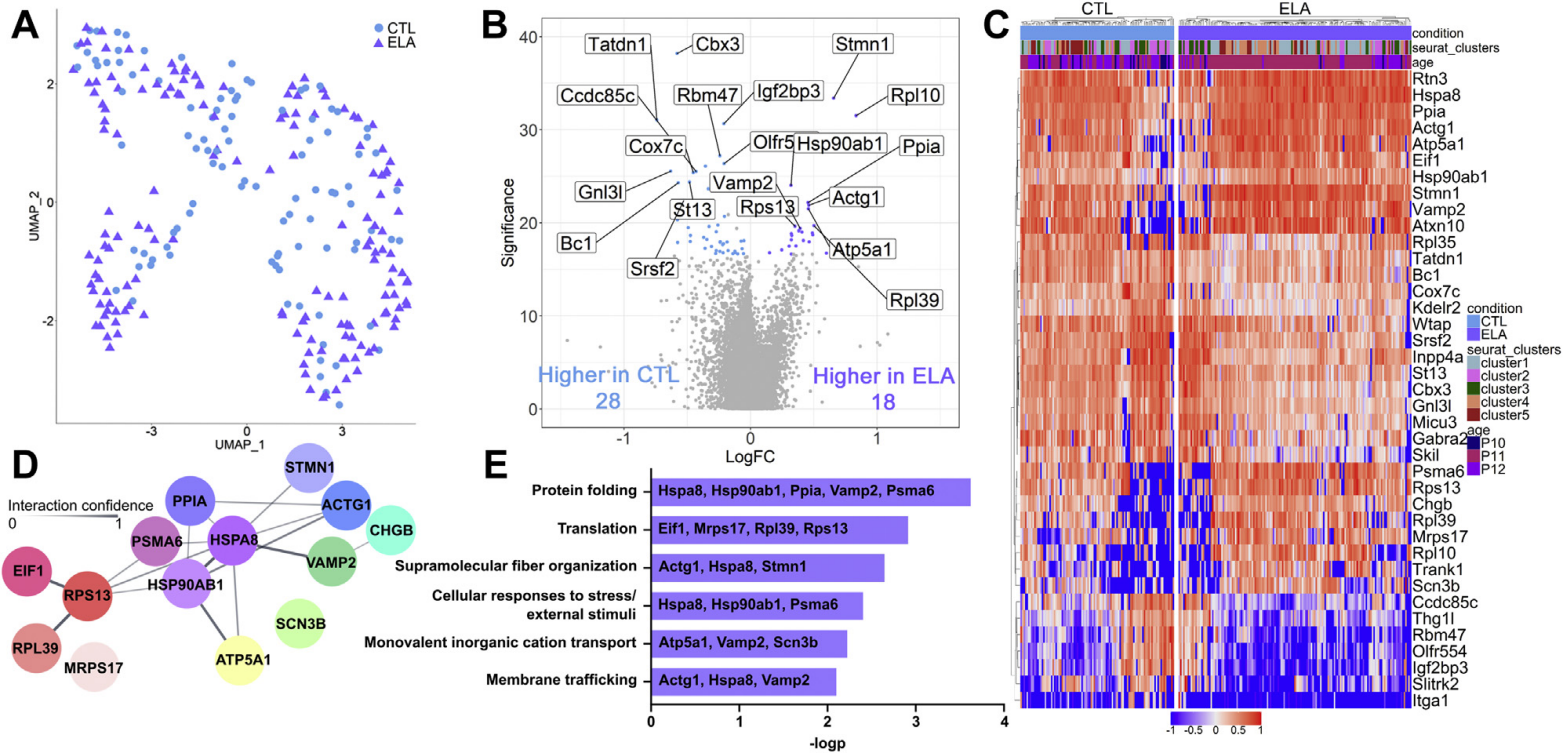
Transcriptomic changes induced by ELA in corticotropin-releasing factor neurons of the paraventricular nucleus. **(A)** Cells from CTL (102 cells) and ELA (152 cells) mice are similarly distributed across the UMAP clusters. **(B)** The volcano plot identifies genes that are significantly enriched (logFC) in CTL (blue) and ELA (purple) cells. **(C)** ELA and CTL cells have different transcriptomic profiles that are independent of age or cluster (logFC by row). **(D)** Predicted protein-protein interaction (Cytoscape) of genes enriched in ELA. Nodes represent proteins and edges represent predicted interaction; line strength indicates confidence of predicted interactions between 0 and 1. Cool colors represent genes associated with neuron activation, and warm colors represent genes associated with translation and protein folding. **(E)** Metascape analysis revealed genes significantly (−logp) associated with predicted pathways as identified by Gene Ontology. CTL, control; ELA, early-life adversity; FC, fold change; P, postnatal day; UMAP, Uniform Manifold Approximation and Projection.

The overall distribution of CRF+ cells within clusters did not distinguish between mice reared under control conditions and those experiencing a week of ELA during a sensitive developmental period (χ^2^_4_ [*n* = 254] = 7.42, *p* = .11) (Figure 2A); there was slight underrepresentation in cluster 2 and overrepresentation in cluster 4 of ELA cells compared with chance (Figure S3A). In contrast, transcriptional analyses of ELA compared with control cells revealed profound changes in the expression of several key genes and gene families (Figure 2B, C). Using a false discovery rate < 0.1, 46 genes were differentially expressed in the CRF+ population of control and ELA mice. Of these, 28 were higher in controls and 18 in ELA cells (Figure 2B).

String network (77) and Gene Ontology enrichment (64) analyses of genes differentially upregulated in ELA mice revealed their predicted protein interactions (Figure 2D) and functional pathway associations (Figure 2E). Genes upregulated after ELA included pathways involved in cellular response to stress and in protein folding (Figure 2D, E). Two genes encoding heat-shock proteins, *Hspa8* and *Hsp90ab1*, which are members of the HSP90 family associated with response to environmental stressors (78) were augmented, as well as *Psma6* and *Ppia*. There was also enrichment of genes associated with regulation of synaptic vesicle content and transport (*Vamp2*, *Chgb*, *Atp5a1*, and *Scn3b*), membrane trafficking (*Atp5a1*, *Vamp2*, and *Scn3b*) and neuronal structure (*Actg1*, *Hspa8*, and *Stmn1*) (Figure 2D, E). String analyses demonstrated predicted protein interactions of the upregulated heat-shock proteins and the products of genes associated with the multiple enriched pathways (Figure 2D). In cells from ELA mice, pathway analysis also confirmed upregulation of translation (*Eif1*, *Mrps12*, *Rpl39*, and *Rps13*) (Figure 2E). We then determined if these gene expression changes, apparent at a single-cell level, could be detected in a tissue block and if they were altered enduringly. We performed quantitative polymerase chain reaction for a subset of these genes using whole PVN punches from adult male mice (Figure S4). Whereas genes associated with neuronal structure (*Stmn1*) were detectably upregulated in the tissue blocks from adult ELA mice, genes associated with response to stimulus (*Psma6, Hsp90ab1*) were not. In contrast to the several gene networks enriched in ELA cells described above, network analyses of genes upregulated in control cells did not reveal significantly interacting genes or enriched pathways.

### Gene Expression Programs Are Differentially Influenced by ELA in Distinct Subpopulations of CRF-Expressing Cells

CRF+ cells in the PVN belong to populations with distinct functional outputs (16,17,26). Therefore, we examined if specific populations of the individually analyzed cells were differentially influenced by ELA. To better identify potential neuronal subpopulations, we first delineated the genes whose expression defined individual cell clusters (Figure 1E). The expression-based clustering identified patterns of gene expression that suggested that clusters 1, 3, and 4 are primarily glutamatergic and clusters 2 and 5 are GABAergic (for detailed summary, see Figure S3B). This cluster analysis was based on CRF+ neurons from both ELA and control populations. Therefore, to exclude the possibility that ELA might modulate the expression state or clustering of the population of PVN CRF cells in the developing mouse, we performed the same cluster analysis on control cells only and determined that segregation of PVN CRF cells into several biologically distinct clusters was not driven by ELA-induced changes to transcription (Figure S5).

We then determined if these expression-defined cell subpopulations were differentially affected by ELA by computing differential expression between ELA and control cells (using a false discovery rate < 0.1) separately for each cluster (Figure S6). Strikingly, differentially expressed genes after ELA were exclusive to cluster 1 (Figure S6A). Genes upregulated in cluster 1 included *Calr*, *Hsbp1, Nnat, Rpl10, Sez6l2*, *Stmn1*, and *Vamp2* (Figure S6A).

### Neurotransmitter Profiles Uncover Novel Populations of PVN CRF Cells That Are Selectively Vulnerable to ELA

**Figure 3:**
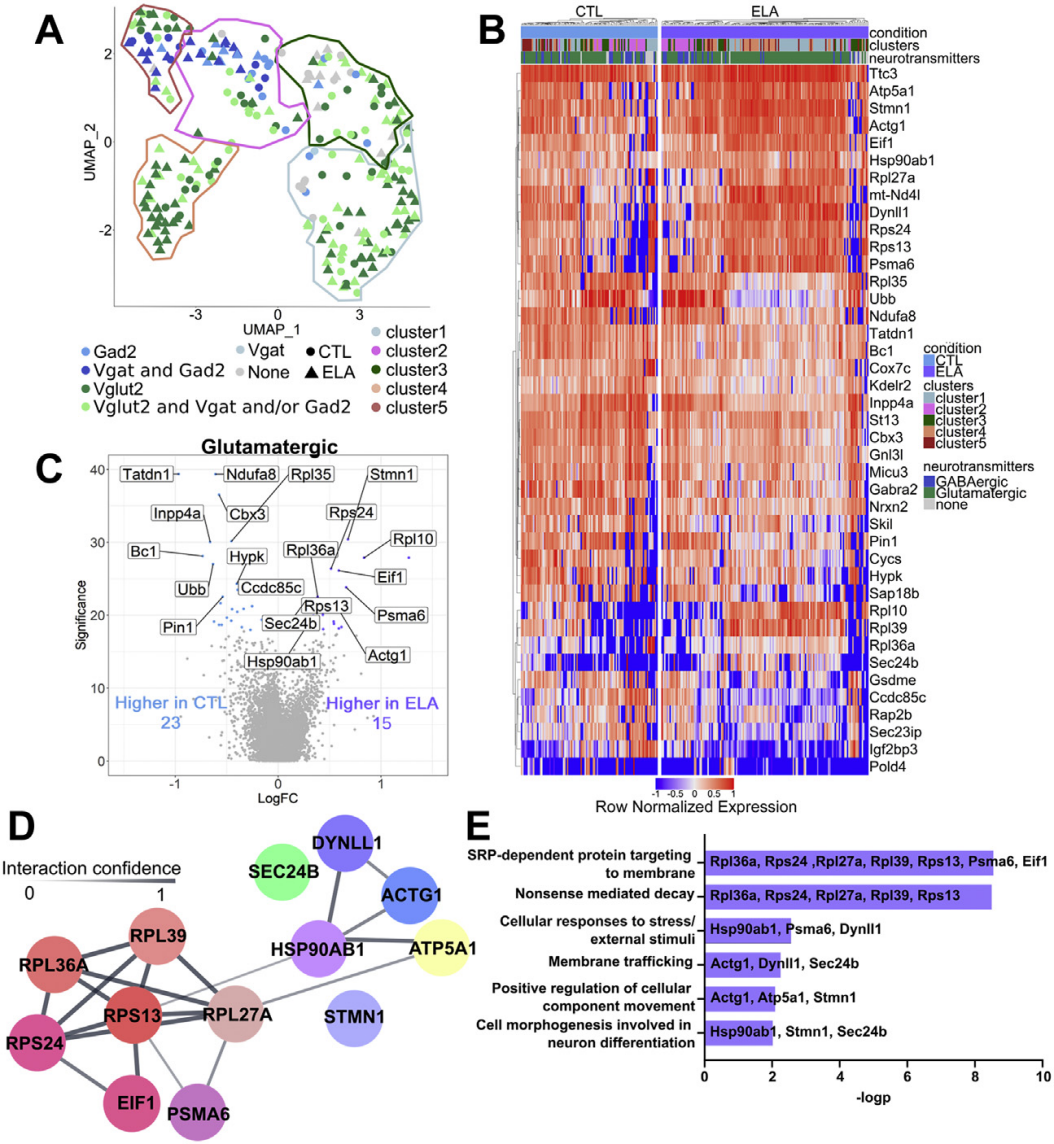
Cell type–specific transcriptomic changes induced by ELA. **(A)** Expression of neurotransmitters overlaid on the UMAP clustering (Seurat clusters 1–5 circled in colored lines, glutamatergic cells expressing *Slc17a6*/VGLUT2 and *Gls* in greens and GABAergic cells expressing *Gad2* and *Slc32a1*/VGAT in blue). **(B)** Normalized expression of top genes in CTL and ELA cells across the two types of clusters. **(C)** Genes with significantly increased gene expression changes comparing CTL and ELA glutamatergic cells. **(D)** Predicted protein-protein interaction (Cytoscape) of genes enriched in ELA. Nodes represent proteins and edges represent predicted interaction; line strength indicates confidence of predicted interactions from 0 to 1. Cool colors represent genes associated with neuron activation, and warm colors represent genes associated with nonsense-mediated decay and protein trafficking. **(E)** Metascape analysis identifies genes significantly (−logp) associated with predicted pathways, which are represented by Gene Ontology terms. CTL, control; ELA, early-life adversity; FC, fold change; GABAergic, gamma-aminobutyric acidergic; UMAP, Uniform Manifold Approximation and Projection.

A canonical characteristic of neurons involves their major neurotransmitters, including glutamate and GABA. The above expression-based clustering suggested that clusters 1, 3, and 4 are primarily glutamatergic and clusters 2 and 5 are GABAergic. Therefore, we determined whether individual CRF+ neurons in developing mouse PVN coexpress either or both neurotransmitters and how the neurotransmitter-defined cell populations related to those defined by agnostic gene expression profiles. We superimposed the Seurat-based clusters onto the expression of glutamatergic markers (the transporter VGLUT2/*Slc17a6* and the enzyme glutaminase/*Gls*) versus GABAergic markers (the synthesizing enzyme GAD2/*Gad2* and the vesicular GABA transporter VGAT/*Slc32a1*). As illustrated in Figure 3A, GABAergic CRF+ cells strongly overlapped with clusters 2 and 5, whereas glutamatergic cells dominated the other clusters (χ^2^_4_ [*n* = 254] = 52, *p* < .00001). The few cells devoid of any neurotransmitter were equally distributed among clusters, suggesting that their expression of neurotransmitter markers simply did not reach detection threshold. Notably, ELA did not significantly change the relative distribution of neurotransmitter-defined subpopulations (χ^2^_3_ [*n* = 254] = 4.351, *p* = .226).

Differential expression analysis between neurons from the ELA and control groups within each neurotransmitter-defined cluster revealed that differentially expressed genes (false discovery rate < 0.1) between control and ELA cells were highly subtype specific (Figure 3B). There were no differentially expressed genes in the GABAergic cluster or that comprised cells expressing no neurotransmitter markers. By contrast, cells within the glutamatergic cluster were transcriptionally modulated by early-life experiences: 23 differentially expressed genes were enriched in control cells and 15 were enriched in ELA glutamatergic cells (Figure 3C).

Of the genes enriched in control glutamatergic CRF+ cells, 14 were also differentially expressed in the global control condition (Figure 2B), including *Cbx3, Cox7c, Gnl3l, Gabra2, Inpp4a*, and *Tatdn1*. Genes enriched uniquely in the control glutamatergic cluster and not in the global control population included *Nrxn2*, *Ubb*, *Cycs, Sec23ip, Sap18b, Pin1*, and *Ndufa8*. Broadly, the genes upregulated in control cells are those that are involved in the use of the electron transport chain and promote growth: *Cox7c* is required in the oxidative phosphorylation pathway for adenosine triphosphate (ATP) generation (79,80); *Ndufa8,* the NADH dehydrogenase ubiquinone 1 α subcomplex, is heavily involved in mitochondrial oxidative phosphorylation and is expressed in the hypothalamus (81); and *Cycs* is the somatic isoform of cytochrome *c* and is upregulated by neural activity (82). The differential expression of the ubiquitin-encoding gene *Ubb* between control and ELA excitatory CRF cells may represent differences in posttranslational modifications (83). Gene Ontology analysis of these genes upregulated in control cells indicated significant enrichment in pathways involved in ATP synthesis–coupled electron transport, positive regulation of the intrinsic apoptotic signaling pathway, and regulation of protein binding representing typical cellular metabolism and activity.

Of the 15 differentially expressed genes enriched in the ELA cells, nine were identified in the overall population analyses (Figure 2B) and six were unique to the glutamatergic cluster: *Rps24, Rpl36a, Sec24b, Dynll1, Nd4l*, and *Rpl27a*. Pathway analysis suggested that these genes are associated with response to environmental stressors, regulation of cellular movement, and upregulation of translation. The protein interactions (Figure 3D) and functional pathway associations (Figure 3E) for genes upregulated in glutamatergic ELA cells indicate similar gene networks and interaction patterns to those of the total ELA CRF cell population, namely overrepresentation of genes associated with response to environmental stressors and neuronal activation. *Hsp90ab* was again a central regulator with predicted associations with gene products involved in protein targeting and nonsense-mediated decay, cellular response to stress, membrane trafficking, and cellular movement and differentiation (Figure 3D, E).

### Novel Populations of Glutamatergic Cells Are Differentially Affected by ELA

**Figure 4:**
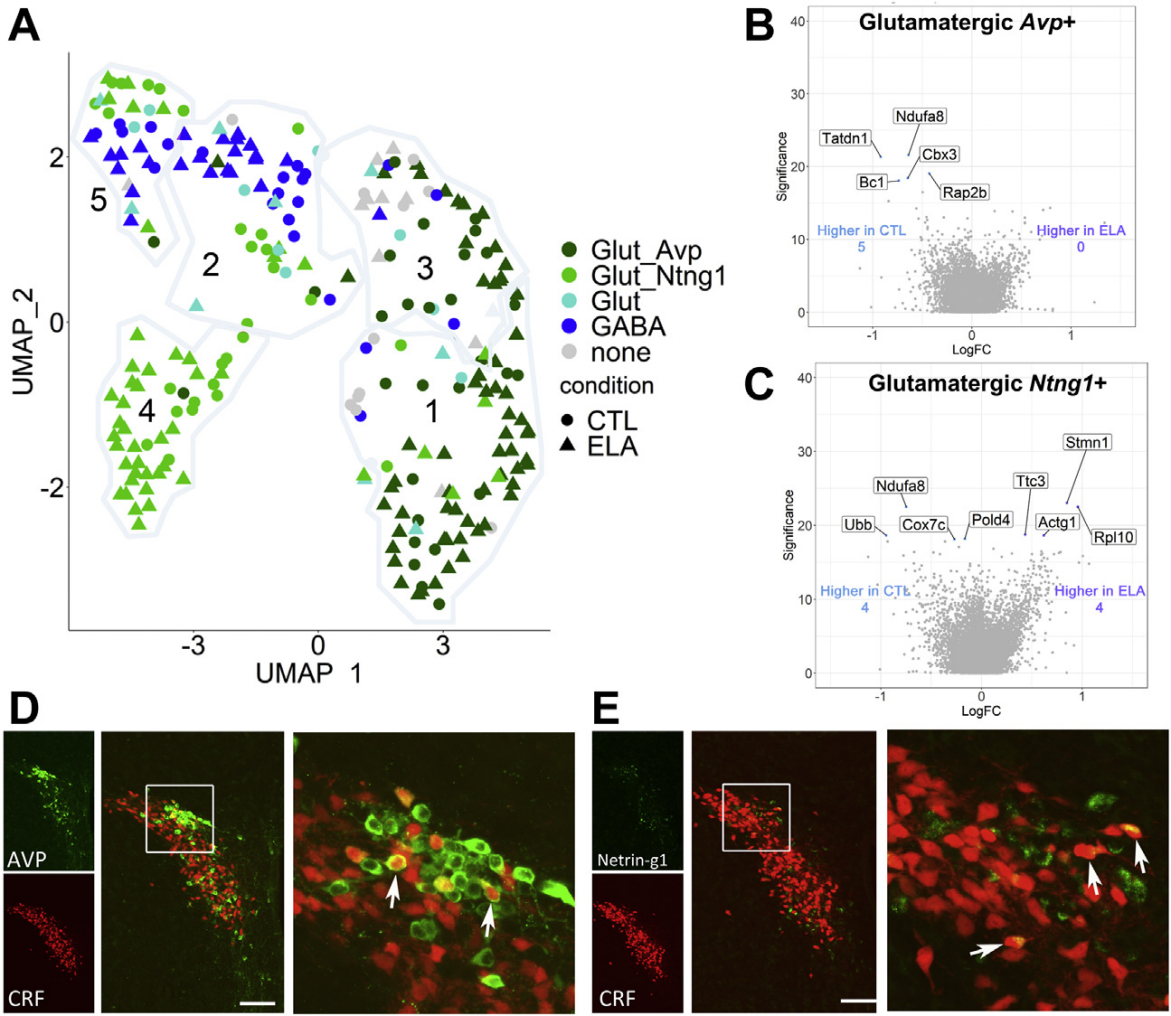
ELA-induced transcriptomic changes are specific to novel subpopulations of CRF-expressing glutamatergic neurons, which are differentially spatially organized in the paraventricular nucleus. **(A)** In CRF-expressing paraventricular nucleus cells, neurotransmitter coexpression with *Avp* or *Ntng1* overlaid on the UMAP clustering demonstrates distinct glutamatergic subclusters. Seurat clusters 1–5 are circled, glutamatergic CRF+ cells expressing *Slc17a6* and *Gls* in are labeled in greens, and GABAergic cells expressing *Gad2* and *Slc32a1* are labeled in blue. **(B, C)** Distinct effects of ELA on gene expression in the *Avp* and *Ntng1* subclusters. Genes with significantly increased logFC in CTL and ELA glutamatergic cells expressing *Avp***(B)** and *Ntng1***(C)** (CTL = blue and ELA = purple). **(D)** Dual immunohistochemistry and confocal microscopy demonstrate the spatial organization of neurons coexpressing CRF promoter–driven tdTomato and AVP. **(E)** Pattern of distribution of paraventricular nucleus neurons coexpressing CRF promoter–driven tdTomato and Netrin-g1. Coexpressing cells are highlighted by white arrows. Scale bar = 20 μM. AVP, arginine vasopressin; CRF, corticotropin-releasing factor; CTL, control; ELA, early-life adversity; FC, fold change; GABA, gamma-aminobutyric acid; UMAP, Uniform Manifold Approximation and Projection.

The neurotransmitter-based segregation of CRF+ PVN cells strongly suggested that glutamatergic cells further belonged to two distinct subgroups (Figure 4A). Therefore, we examined if the impact of ELA on gene expression was selective to one of these subpopulations. The two subclusters of glutamatergic CRF cells were distinguished by their expression of *Avp*, the gene encoding vasopressin, or *Ntng1*, a cellular adhesion molecule important for axon guidance (84, 85, 86) (Figure 4A). Indeed, differential gene expression between ELA and control neurons was distinct and nonoverlapping in these two subsets: in the *Avp*-positive subcluster (Figure 4B), differentially expressed genes were specifically enriched in control cells and included *Tatdn1*, a long noncoding RNA (87); *Ndufa8*, which encodes a subunit of NADH dehydrogenase, part of the electron transport chain (83); *Cbx3*, a member of the heterochromatin protein 1 family that stimulates cellular differentiation (88); the prosurvival gene *Rap2b* (RAP2B) (89); and *Bc1,* which is important for translation (90). The functions of these genes enriched in control *Avp* CRF+ cells are required for normal cellular maturation and activity.

Four genes were enriched in the control glutamatergic *Ntng1* subcluster (Figure 4C), including *Ubb*, *Ndufa8*, *Cox7c*, and *Pold4*, and the latter was uniquely identified in this subcluster analysis. *Ndufa8* and *Cox7c* are required for the electron transport train (79, 80, 81). *Ubb* encodes ubiquitin, which controls protein targeting and degradation and has a role in controlling the stress response (91,92). *Pold4* encodes a subunit of DNA polymerase and is critical for DNA repair (93). Notably, several genes (*Stmn1*, *Rpl10*, and *Actg1*) with important neuronal growth functions were uniquely downregulated in the control glutamatergic *Ntng1*-positive subcluster (enriched in ELA cells) (Figure 4C).

### Novel Populations of PVN CRF+ Neurons Are Spatially Organized

The discovery of novel populations of CRF+ glutamatergic cells in the PVN, which are differentially influenced by ELA, relied on single-cell transcriptomics and cluster and neurotransmitter analyses. To better characterize them, we determined their physical distribution in the PVN using immunohistochemistry. As shown in Figure 4D, a subset of PVN neurons in a P10 mouse expressing tdTomato under the CRF promoter coexpressed arginine vasopressin and resided primarily in the dorsal PVN. By contrast, cells expressing CRF+ netrin-g1 were distributed throughout the PVN, without apparent spatial organization (Figure 4E).

### Transcriptional Changes Provoked by ELA Herald Enduring Alterations of Behavioral and Hormonal Responses to Stress

**Figure 5:**
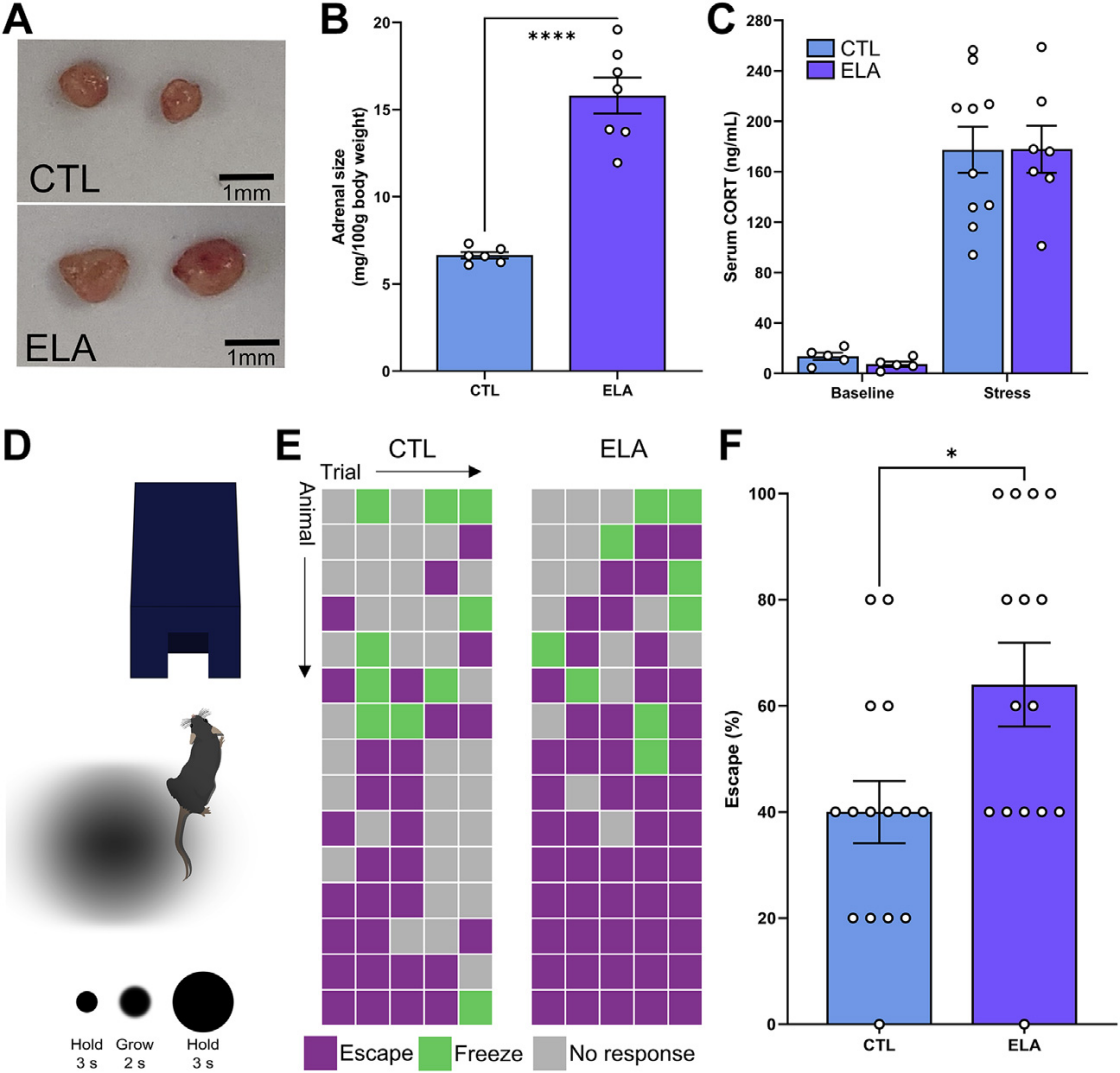
ELA-induced transcriptional changes in corticotropin-releasing factor–expressing paraventricular nucleus neurons herald enduring augmentation of neuroendocrine and behavioral responses to stress. **(A, B)** ELA causes significant increase in adrenal size (*n* = 6–7 per group), a hallmark of a chronic stress state. **(C)** Hormonal responses to acute stress, measured as serum corticosterone at baseline or after 1 hour of multiple acute stress, are not influenced by ELA (*n* = 5–10 per group). **(D)** Schematic of looming disk threat stimulus test. **(E, F)** Results from each of the five looming threat stimulus trials for each mouse identify a significant increase in escape behaviors in ELA mice (*n* = 15 per group). Bars represent mean ± SEM; circles represent individual mice. CTL, control; CORT, corticosterone; ELA, early-life adversity.

To determine the potential functional long-term significance of the transcriptional changes induced by ELA in glutamatergic PVN CRF cells, we tested stress-related behavioral and hormonal parameters in adult ELA and control mice. We assessed measures of chronic increases of CRF release and the release of its downstream stress hormones using adrenal gland weights (94). We also measured hormonal responses to acute stress. Adrenals of adult ELA mice were significantly heavier than those of control mice (*t*_11_ = 4.1, *p* = .002), an effect that persisted when corrected for body weight (*t*_11_ = 8.1, *p* < .0001) (Figure 5A) (37,43), indicating chronically heightened stress reactivity (94). After acute stress (67,68), serum corticosterone levels rose dramatically (*F*_1,23_ = 89.11, *p* < .0001), with no main effect of ELA (*F*_1,23_ = 0.03, *p* = .87) and no stress by ELA interaction (*F*_1,23_ = 0.03, *p* = .85) (Figure 5B). Together, these experiments indicate that transcriptional changes induced by transient ELA result in an enduring and robust stress phenotype. Whereas responses to acute stress do not distinguish adult ELA from control mice, ELA mice release much more hypothalamic CRF and pituitary adrenocorticotropic hormone chronically (33), resulting in adrenal hypertrophy, the hallmark of a chronic stress state.

The functional role of PVN CRF cells in orchestrating behavioral responses to stress has not been fully elucidated. A requirement of these neurons in behavioral responses to threat was recently shown (8), and so we tested ELA and control mice in a test eliciting threat responses, the looming shadow task (66). Escape behaviors of ELA mice were significantly higher than control mice (*t*_22_ = 2.449, *p* = .02) (Figure 5C, D), suggesting that ELA-induced transcriptional changes may contribute to altered adult behavioral responses to stressful threats.

## Discussion

Several principal findings emerge from these studies, which use single-cell transcriptomics analysis of CRF-expressing PVN neurons and determine the impact of adverse/stressful early-life experiences on gene expression patterns. CRF-expressing neurons in PVN cluster into distinct populations by both their gene expression profiles and their neurotransmitter phenotype, and these populations are distinctly spatially organized. ELA modifies gene expression patterns, affecting transcriptional programs of neuronal development and differentiation and enhancing gene families involved in responses to stress and inflammation. The use of single-cell transcriptomics reveals that ELA affects gene expression profiles in a cell type–specific manner, with unique influence on different clusters and subpopulations of CRF neurons. Finally, the transcriptional changes identified immediately after ELA herald significant enduring disruptions of hormonal and behavioral stress responses.

### PVN Contains Discrete, Molecularly Defined Clusters of CRF Cells

Clustering using Seurat identified differences in gene expression profiles of CRF-positive PVN neurons, which may be consistent with prior work (24, 25, 26,95). Clusters 1 and 3 had increased expression of *Avp* and steroid hormone receptors, whereas the transcriptomic profile of cluster 5 suggests that these represent a small population of cells that coexpress GABA and CRF. Previous studies suggested that molecularly defined clusters were not spatially organized within the PVN yet may define cells performing different functions or sampled at differing stages of development (26,96). Here, we identify novel populations of CRF+ cells, with distinct spatial organization and differential responses to ELA. To fully elucidate transcriptomic and functional phenotypes of these patterns, future work will harness technologies that allow for analysis of greater cell numbers and those that provide spatial resolution.

### ELA Enriches Genes Associated With Neuronal Maturation, Differentiation, and Stress

Analysis of the entire CRF cell population revealed specific changes in ELA and control cells’ gene expression profiles. Specifically, while control cells engage in typical growth/metabolism functions, ELA cells are enriched for genes important for neuronal maturation, differentiation, and mitigating neuronal stress. Genes such as *Cox7c* and *Micu3* that are required in the oxidative phosphorylation pathway for ATP generation (79,80) were enriched in control cells, as was *Cbx3*. A gene important for neural differentiation, *Cbx3* increases expression of neuronal genes and inhibits expression of genes specific to other fates (88). Thus, its downregulation in ELA cells suggests a potential adaptive neuronal dedifferentiation of CRF cells consequent to ELA, similar to observations in the hippocampus (33). A neuronal fate is highly expensive metabolically, requiring energy-intensive maintenance of membrane potential and neuronal firing. Dedifferentiation may save the cell from death in stressful conditions.

Contrary to control cells, ELA cells have expression profiles consistent with response to stress and neuronal activation (Figure 2D, E). For example, the HSP90 family of heat-shock proteins facilitates steroid hormone receptor function, maintains the glucocorticoid receptor in the high-affinity binding state, and enables translocation to the nucleus (97,98). Upregulation of *Hsp90ab1* in ELA mice may be important for signaling cascades responsible for cellular adaptation to stress. Future work will use single-cell resolution in situ experiments to fully validate if these effects are enduring and characterize spatial organization of the clusters.

### Genes Upregulated in ELA Hypothalamic CRF Cells Are Those That Typically Contribute to Increased Activity in Glutamatergic Excitatory Neurons

The clustering analyses here highlight the complexity of PVN CRF-expressing cells (Figure 1E) and identify neuronal populations beyond those described previously. Specifically, we orthogonally categorized cells also by neurotransmitter expression patterns (Figure 3A). As described previously (9,22,60,99), PVN CRF-expressing cells were predominately glutamatergic, and the effects of ELA were only observed in these glutamatergic CRF+ cells. Notably, the transcriptomic changes following ELA observed here take place in immature mice. Thus, it is possible that analyses of neurons from mature, adult mice may reveal alterations in other populations (e.g., GABAergic cells).

What might the ELA-induced changes in gene expression patterns of glutamatergic CRF cells signify? Following ELA, there is a significant increase in functional excitatory synapses on PVN CRF cells (30,33). This may increase their metabolic demand and their response to input from other brain regions that process stress. Our results support both of these possibilities: first, differentially expressed genes enriched in glutamatergic ELA cell clusters (*Dynll1*, *Nd4l*, *Rpl27a*, *Rpl36a*, *Rsp24*, and *Sec24b*) largely encode proteins required for transcription and translation complexes (100, 101, 102), and Gene Ontology identifies nonsense-mediated decay as a significantly upregulated pathway after ELA. Nonsense-mediated decay involves targeted degradation of messenger RNA in conditions of cell stress and apoptosis (103).

Notably, among the two novel subclusters of glutamatergic cells (*Avp+* or *Ntng1+*) (Figure 3), all ELA-induced gene expression enrichment occurred in the *Ntng1* group driving global transcriptomic changes after ELA. Netrin-G proteins regulate synapses. Loss of netrin-G1 interaction with its ligand (NGL-1) reduces excitatory synaptic plasticity (104). Although the function of the *Ntng1+* PVN CRF+ cluster is unknown, ELA-provoked changes in these cells are congruent with—and may mediate—the aberrant increase in excitatory synapses onto ELA CRF+ cells. Finally, the gene expression changes support increased activity of ELA PVN CRF cells, consistent with their increased excitatory innervation as described previously (30,33). If persisting in the adult, such changes may enhance cellular activity in response to stressors over the life span, resulting in increased neuroendocrine responses and adrenal size, often associated with chronic stress. Functionally, activity of CRF+ PVN cells is associated with responses to threat (8). Accordingly, we observed augmented escape behaviors of ELA mice compared with control mice, suggesting augmented activity of PVN CRF cells (8).

### Conclusions

In conclusion, the use of single-cell transcriptomics enables an unprecedented level of insight into the diverse CRF-expressing neuronal populations within the PVN and the altered gene expression patterns provoked by ELA in these neurons. We highlight a new level of transcriptomic heterogeneity of PVN CRF cells and segregate them into biologically meaningful clusters with distinct spatial organization and differential vulnerability to ELA. Understanding which cell types undergo transcriptional programming in response to early environmental signals and how these experiences are encoded transcriptionally is vital for identifying novel targets for mitigating the enduring adverse consequences of ELA.

## Supplementary Material

Supplementary Material

Key Resources Table
