# Supplementary material for: Single-Cell Transcriptional Changes in Hypothalamic Corticotropin-Releasing Factor–Expressing Neurons After Early-Life Adversity Inform Enduring Alterations in Vulnerabilities to Stress
Source: Biol Psychiatry Glob Open Sci. 2021 Dec 23;3(1):99–109. doi: 10.1016/j.bpsgos.2021.12.006 (PMC9874075; doi:10.1016/j.bpsgos.2021.12.006)
Supplement: Supplementary Material [file mmc1.pdf]

Single-Cell Transcriptional Changes in Hypothalamic Corticotropin-Releasing Factor Expressing Neurons After Early-Life Adversity Inform Enduring Alterations in Vulnerabilities to Stress

Supplementary Information

Supplementary Methods

Early-life adversity paradigm cages with limited bedding and nesting (LBN). We imposed ELA on neonatal mice using simulated poverty by limiting nesting and bedding materials in cages during the early developmental period as described previously (1–3). For RNA sequencing, pups remained on the LBN paradigm until tissue was collected P10-P12. For experiments in adulthood, experimental groups were transferred to standard cages on P10 and were weaned on P21. Animals were housed by sex, with littermates. For the LBN group, a plastic-coated mesh platform was placed ~2.5cm above the floor of a standard cage. Cobb bedding was reduced to cover the cage floor sparsely, and one-half of a single nestlet was provided for nesting material on the platform. Control dams and litters resided in standard cages containing ample cob bedding and one whole nestlet for nesting. This paradigm causes maternal care to be fragmented and unpredictable (4), provoking chronic stress in the pups (5,6). Control and experimental cages were undisturbed during experimental period housed in temperature-controlled rooms (22°C).

Single cell preparation

Dissection. P10-P12 male pups were killed via decapitation and brains were removed immediately on ice (CTL: *n*=50 pups from 14 litters, and ELA: *n*=38 pups from 10 litters). The brain was trimmed to a smaller block containing hypothalamus and was placed into a slush of EBSS (NaCl 116 mmolL-1, KCl 5.4 mmolL-1, CaCl2 1.8 mmolL-1, MgSO4.7H2O 0.4 mmolL-1, NaHCO3 26.2 mmolL-1, Glucose 5.5 mmolL-1). The block was then sliced on a vibratome to obtain at 1.5mm slice posterior of the anterior commissure. The slice was then trimmed under a dissection microscope to remove thalamus and cortex.

Dissociation. The trimmed slices were placed immediately into papain (20 units/mL; Worthington, NJ, USA) and gently triturated ~15 times with a pipette to break up tissue before being placed on a heated (37C) orbital shaker for 30 minutes. Digested tissue was then homogenized by triturating with pipette ~ 50 times until there were no visible tissue chunks. Homogenate was spun at 500 x g for 15 minutes at room temperature. Supernatant was removed and cells were resuspended in 500uL 2% FBS in PBS.

FACS. Samples were run on the BD FACSaria fusion (BD Biosciences, NJ USA). Immediately prior to sort, cell suspension was run through a 70uM filter and washed with 500uL 2% FBS in PBS. Cells were sorted into 8 well strip tubes and immediately spun down at 4C and frozen on dry ice.

qPCR analysis of PVN punches

PVN punch collection. Punches were collected from 6-month-old male mice (n= 7 CTL and 10 ELA from two litters each). Dorsal hypothalamus containing PVN was extracted using the Palkovits Punch technique. Briefly, 3 x 200um fresh-frozen brain sections were cut on a cryostat (Leica 3050s) containing PVN (Coordinates $^{-}$0.6 - $^{-}$1.2 Bregma) and extracted with a 1mm tissue corer and stored at -80°C until RNA extraction.

qRT-PCR. Adult male mouse hypothalamic punches were processed for total RNA isolation using the Direct-zol RNA purification kit (Zymo Research, Irvine). 100 ng of total RNA was reverse-transcribed using the Transcriptor first-strand cDNA synthesis kit (Roche, IN) with oligo d(T) and random hexamer primers. Each cDNA sample was run in triplicates using FastStart Essential Green Master (Roche, IN) on a Roche Lightcycler 96 system. Data were quantified using the 2^-ΔΔCt method with 18s as housekeeping gene. Primer sequences are provided as follows:

Gene	Forward	Reverse
**18S**	GGGAGCCTGAGAAACGGC	GGGTCGGGAGTGGGTAATTT
Stmn1	TCGGTGTGCTGGGTTAATGG	GGAAGAAGCCGCCACATACT
Psma6	AACGGAAAGCATTGGCTGTG	CATAGCGTGCCCTCTGTACC
Hsp90ab1	AGATTCCACTAACCGACGCC	CTGGGTCTCCTTCATGCGAG

Immunofluorescence staining. P10 male pups were euthanized with sodium pentobarbital and transcardially perfused with ice-cold phosphate-buffered saline (PBS; pH=7.4) followed by 4% paraformaldehyde in 0.1M sodium phosphate buffer (pH=7.4). Perfused brains were post-fixed in 4% paraformaldehyde in 0.1 M PBS (pH = 7.4) for 4-6 hr, before cryoprotection in a 25% sucrose solution. Brains were frozen, then sectioned coronally into 20-µm-thick slices (1:6 series of the PVN) using a Leica CM1900 cryostat (Leica Microsystems, Wetzlar, Germany). Immunofluorescence staining was performed on brain sections derived from P10 male tdTomato-Crh (*Crh*-IRES-Cre;Ai14) transgenic mice as described previously (7,8). Briefly, after several washes with PBS containing 0.3% Triton X-100 (PBS-T, pH 7.4), sections (20 µm) were treated with 0.3% H_2O_2/PBS for 30 min, then blocked with 5% normal goat serum (NGS) for 30 min to prevent non-specific binding. After rinsing, sections were incubated for 3 days at 4°C with rabbit anti-AVP (1:2,000, Sigma, PC234L) or rabbit anti-Ntng1 (1:1,000, ThermoFisher, PA5-30447) in PBS containing 1% BSA, and washed in PBS-T (3 × 5 min).

Immunoreactivity was visualized using anti-rabbit IgG conjugated to Alexa Fluor 488 (1:400, Invitrogen) for 2 hrs (RT).

Confocal imaging. Brain sections from the PVN (equal to AP -0.46 mm to -0.94 mm in the adult, relative to the Bregma) were subjected to confocal imaging (LSM 510, Zeiss). Virtual z-sections of 1 µm were taken with an Apochromat 63× oil objective (numeric aperture 1.40). Image frame was digitized at 12-bit using a 1024 x 1024 pixel frame size. To prevent bleed-through in dual-labeling sections, images were scanned sequentially by two separate excitation laser beams: an Argon laser at a wavelength of 488 nm and a He/Ne laser at 543 nm. Z-stack reconstructions and adjustments of image contrast were performed using ImageJ (version 1.41).

Tests of CRH+ PVN cell function

Looming shadow task. The looming shadow task is largely dependent on the CRH neurons of the paraventricular nucleus (PVN) of the hypothalamus (9). The entire task was performed in the dark phase and as described previously (10). Briefly, male mice were initially habituated to an arena (L33xW18xH16 cm) containing a shelter (L14xW9xH7 cm with a H7xW4 cm opening) covered with an LED monitor (DELL model no. S2316H; 58.4cm; resolution: 1920 x1080 at 60Hz) displaying a grey (#3B3838) background for 15 minutes. Following habituation, a stimulus is presented to the animal from the monitor above which consisted of a black (#000000) expanding disk in the grey background (starts small at ~2.5cm in diameter, holds for three seconds before growing for two seconds to 10x its size and holds for another 3 seconds), which was repeated 5 times with at least 1 minute between stimuli. The mouse's response to the looming stimulus was scored (eg. no response, freezing, escape) both live and on recording by an independent experimenter. Percentage escape was calculated using (number of escapes/number of total trials)*100. Mice were aged-matched 2-8 month old, CTL: n=15 animals from 4 litters, and ELA: n=15 animals from 5 litters.

Stress in adulthood. Responses to acute stress were determined using multiple acute concurrent stresses (11,12) imposed for one hour. This paradigm involves exposing male mice to simultaneous physical, emotional, and social stresses and is described in detail at Bio-protocol (11) and has been utilized in other studies (12–15). Briefly, mice were individually restrained in a ventilated 50mL plastic tube. Two to six mice were placed in a cage atop a laboratory shaker in a room with loud (90dB) rap music and bright lights for one hour. Animals were perfused immediately post-stress and blood was collected through cardiac puncture prior to perfusion and samples were left to clot at room temperature for 30 minutes.

Corticosterone assay. Baseline blood was collected from adult male mice through cardiac puncture prior to perfusion and samples were left to clot at room temperature for 30 minutes. Following centrifugation at 1100 x g for 15 minutes serum was collected at stored at -20°C. Corticosterone in the serum was analyzed using the Corticosterone EIA Kit (Cayman Chemical Company, MI, USA) according to the manufacturer's instructions. Post-stress serum was collected directly following the stress. Optimal serum dilutions were previously established in the lab and a 1 in 50 dilution for basal levels and 1 in 500 for post-stress serum was analyzed in duplicate**.** Animals were aged-matched 2–8-month-old, CTL baseline: *n*=5 animals from 1 litter, CTL stress: *n*=10 animals from 3 litters, ELA baseline: *n*=5 animals from 2 litters, and ELA stress: *n*=7 animals from 2 litters.

Adrenal gland collection. In a separate cohort of male mice, adrenals were collected following a lethal injection (Euthasol solution; ~488 mg/kg pentobarbital sodium and ~63 mg/kg 175 phenytoin sodium, intra-peritoneally). Gross dissection was performed to isolate the left and right adrenals from the surrounding tissue and were weighed together. Adrenal size is expressed as a function of body weight collected at the time of euthanasia. Animals were aged-matched 2–8-month-old, CTL: *n*=6 animals from 2 litters, and ELA: *n*=7 animals from 3 litters.

Supplementary Tables and Figures

Batch ID	Condition	Postnatal Day	# of Animals	Av. Weight (g)	# of cells
Batch-19	Ctrl	10	2	5.35	9
Batch-20	Ctrl	12	2	5.7	8
Batch-21	Ctrl	12	3	6.13	26
Batch-23	Ctrl	11	5	5.78	3
Batch-25	Ctrl	11	3	7.2	21
Batch-26	Ctrl	10	3	5.1	14
Batch-28	Ctrl	12	5	6.22	9
Batch-29	Ctrl	12	3	4.6	13
Batch-31	Ctrl	10	3	6.4	3
Batch-32	Ctrl	12	4	6	23
Batch-33	Ctrl	12	5	6.7	22
Batch-34	Ctl	12	4	5.3	21
Batch-36	Ctl	12	4	6.6	15
Batch-38	Ctrl	11	4	5.2	51
Batch-L7	LBN	12	4	5.45	15
Batch-L9	LBN	12	4	5.2	2
Batch-L10	LBN	12	4	5.9	25
Batch-L11	LBN	10	5	5.14	35
Batch-L12	LBN	11	5	4.1	27
Batch-L13	LBN	11	4	5.7	21
Batch-L14	LBN	12	3	5.5	4
Batch-L15	LBN	12	1	3.7	5
Batch-L16	LBN	11	5	3.8	70
Batch-L17	LBN	11	3	5.1	69

Supplementary Table S1. Details of animals used for single-cell sequencing. Batch ID represents individual litters.

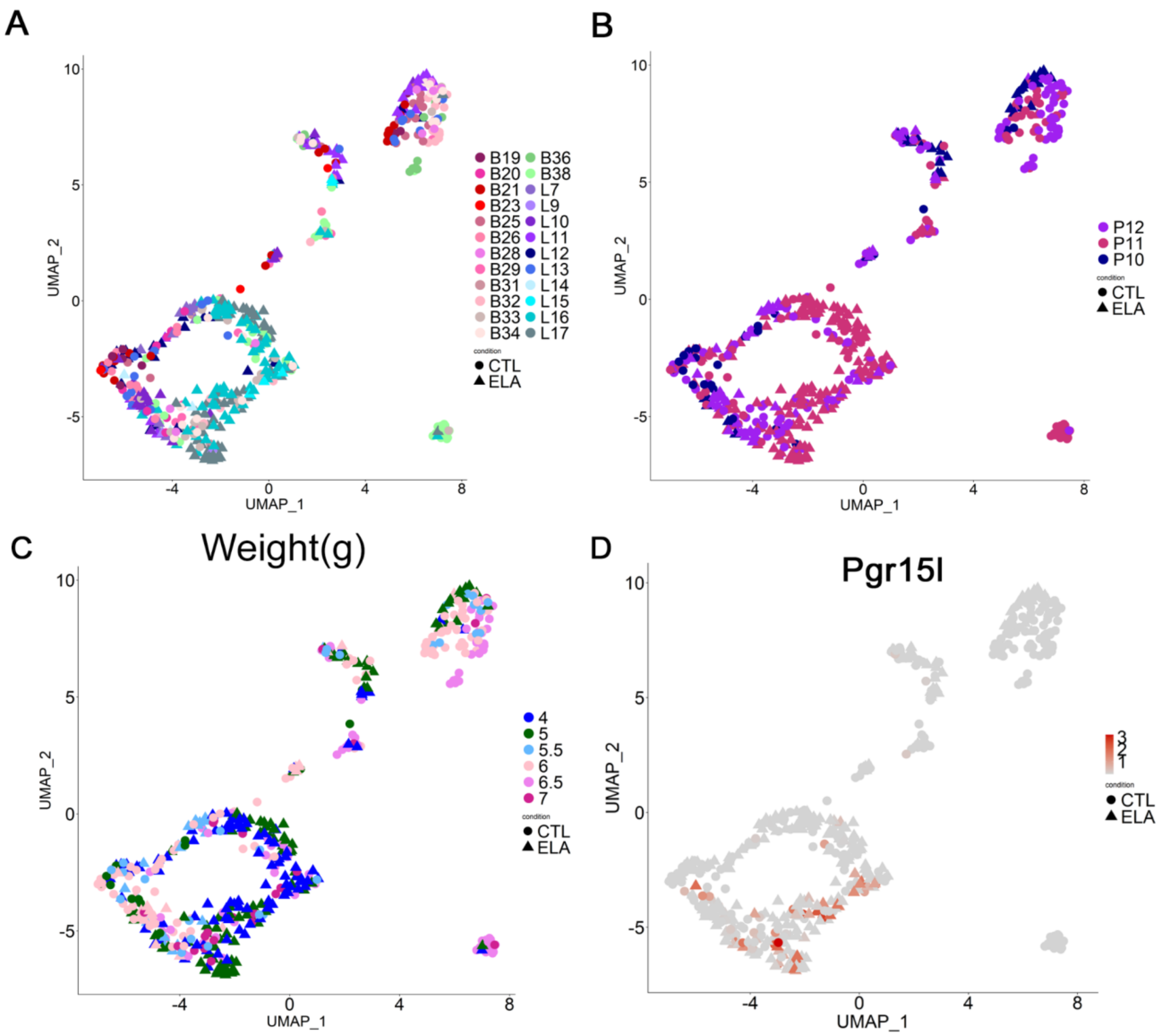

Supplementary Figure S1. Characterization of batch, age, weight, and non-PVN marker *Pgr15l* expression of 511 cells (A) UMAP characterization of 24 batches across 511 cells to determine little or no batch effect between sorts. (B) UMAP characterization of age the cells were harvested between postnatal day 10 - 12. (C) UMAP characterization of weight in grams of mice on the day cells were harvested. (D) Heatmap overlay of non-PVN *Pgr15l* expression over the UMAP of 511 cells to determine cell filter for *Pgr15l* expression. All UMAPs were generated using Seurat v3.

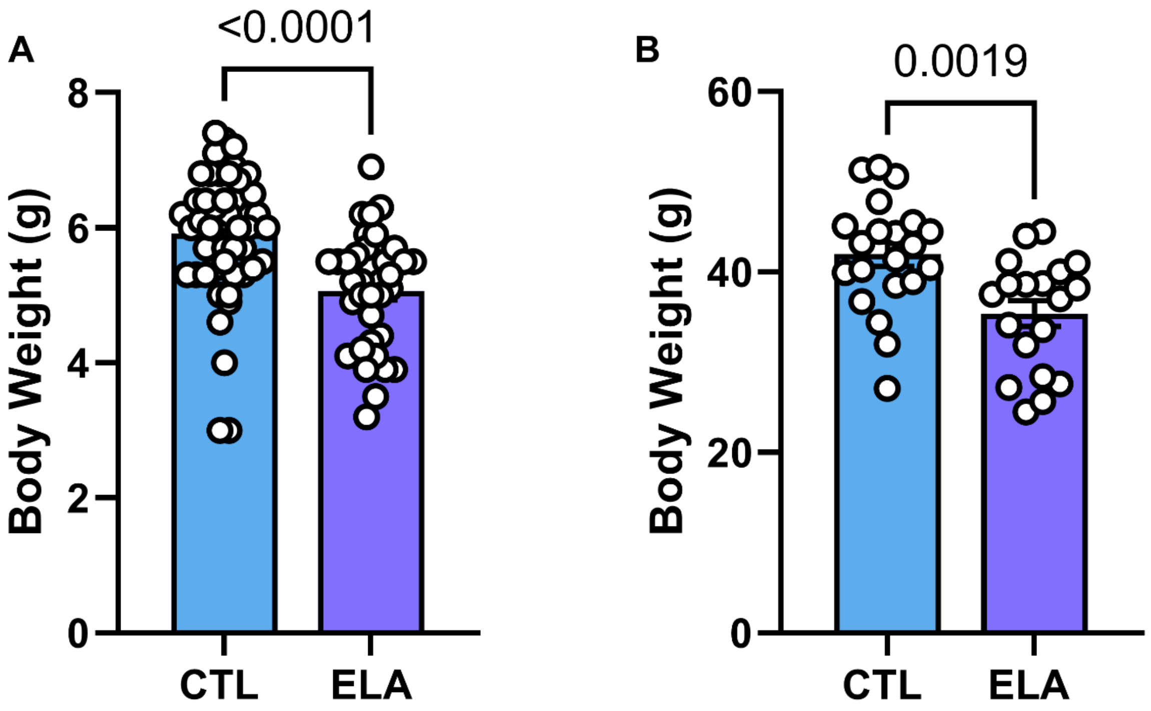

Supplementary Figure S2. Effect of ELA on body weight. (A) Body weights at time of collection for sequencing, there was a significant effect of ELA on body weight ($t_{(86)}$=4.3, p<0.0001) at PND 10-12. (B) Body weights at time of adrenal or CORT collection. In these cohorts, body weight is lower following ELA ($t_{(38)}$=3.3, p=0.002) at 2-8 months of age. Dots represent individual mice; bars represent mean ±SEM.

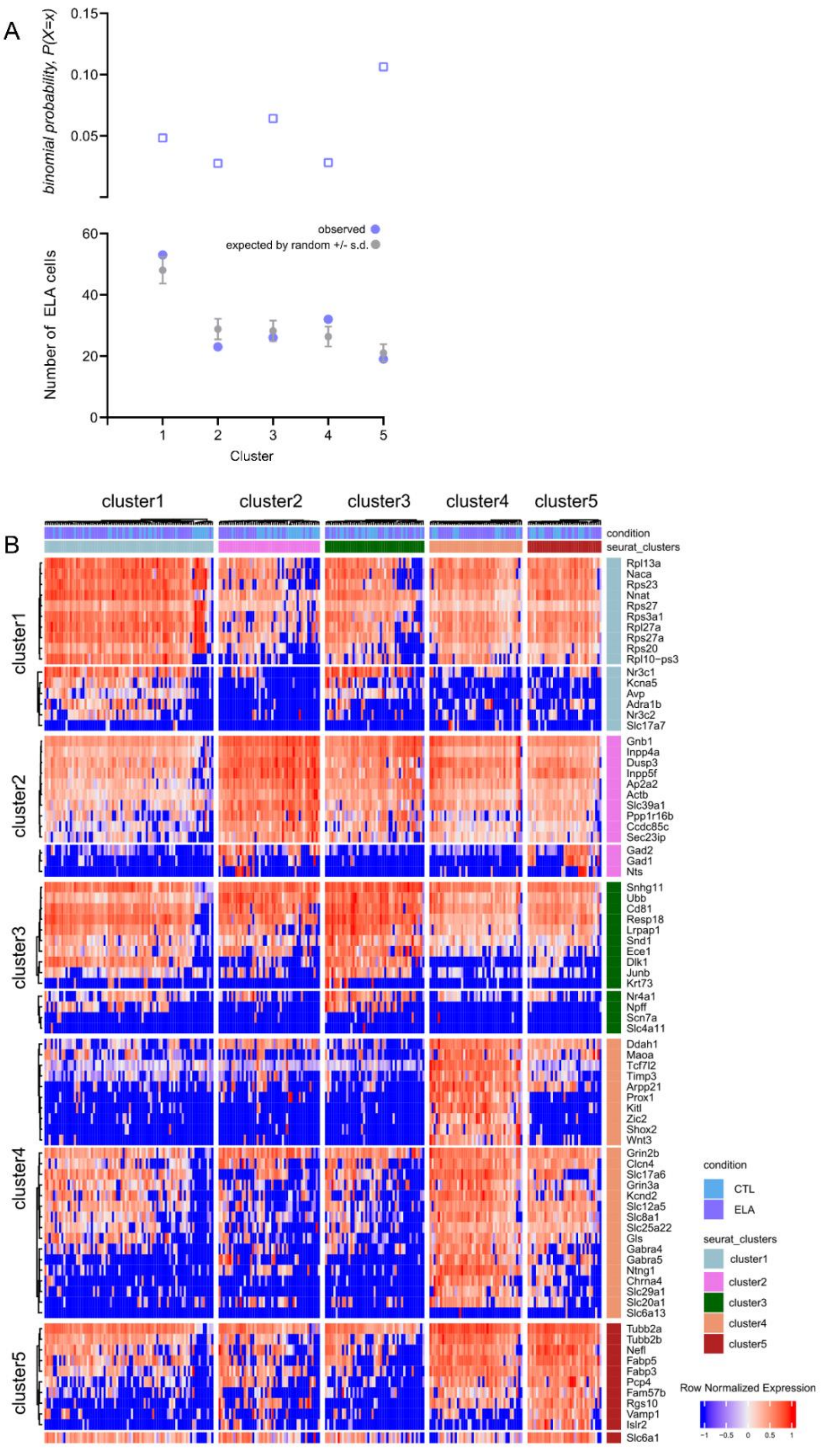

Supplementary Figure S3. Distribution of cells and genes between clusters. (A) The observed number of ELA cells in each cluster compared to what would be expected at random according to binomial distribution and the probability of this occurring by chance. (B) Top 10 genes enriched in each

cluster (top panels for each cluster) and identified genes of interest (bottom panels for each cluster), row log normalized. Genes enriched in cluster 1 included *Avp* (Arginine Vasopressin), the gene encoding the stress-related neuropeptide vasopressin, as well as the stress-steroid receptors *Nr3c1* (mineralocorticoid receptor, MR) and *Nr3c2* (glucocorticoid receptor, GR), *Adra1b* (alpha-adrenergic receptor 1B), and ion channels/transporters including *Kcna5* (Kv1.5, Potassium Voltage-Gated Channel Subfamily A Member 5) and *Slc12a7* (KCl cotransporter 4, KCC4)*.* Cluster 2 was enriched with genes associated with GABAergic neurotransmission including *Gad1* (Glutamate Decarboxylase 1) and *Gad2* (Glutamate Decarboxylase 2) and also expressed *Nts* (Neurotensin)*.* Similar to cluster 1, cluster 3 cells had enriched expression of *Avp*, the steroid receptor *Nr4a1* (Nuclear Receptor Subfamily 4 Group A Member 1) as well as ion channels/transporters including *Scn7a* (Nav2.1, Sodium Voltage-Gated Channel Alpha Subunit 7) and *Slc4a11* (Bicarbonate Transporter Related Protein 1). Cluster 3 was also characterized by high expression of the vasopressin secretion associated gene *Npff* (Neuropeptide FF-Amide Peptide Precursor). Cells in cluster 4 had the most diverse expression profile including high expression of *Ntng1* (Netrin G1), markers of glutamatergic neurotransmission such as *Slc17a6* (vGLUT2) and *Gls* (Glutaminase), and the glutamate receptors *Grin3a* (Glutamate Ionotropic Receptor NMDA Type Subunit 3A, GluN3A) and *Grin2b* (Glutamate Ionotropic Receptor NMDA Type Subunit 3B, GluN3B), the GABA-A receptors *Gabra4* (Gamma-Aminobutyric Acid Type A Receptor Subunit Alpha4) and *Gabra5* (Gamma-Aminobutyric Acid Type A Receptor Subunit Alpha5), the cholinergic receptor *Chrna4* (Cholinergic Receptor Nicotinic Alpha 4 Subunit)*,* and multiple ion channels/transporters (*Kcnd2,* Potassium Voltage-Gated Channel Subfamily D Member 2; *Clcn4,* Chloride Voltage-Gated Channel 4; *Slc29a1,* ENT; *Slc20a1,* Pit-1; *Slc8a1,* NCX1; *Slc12a5,* KCC2; *Slc25a22,* Mitochondrial Glutamate Carrier 1; and *Slc6a13,* GABA Transporter 2). Cluster 4 also had high expression of *Maoa* (Monoamine Oxidase A)*.* Cluster 5 had the fewest differentially expressed genes and was specifically characterized by the expression of the GABA transporter *Slc6a1* (GABA Transporter 1).

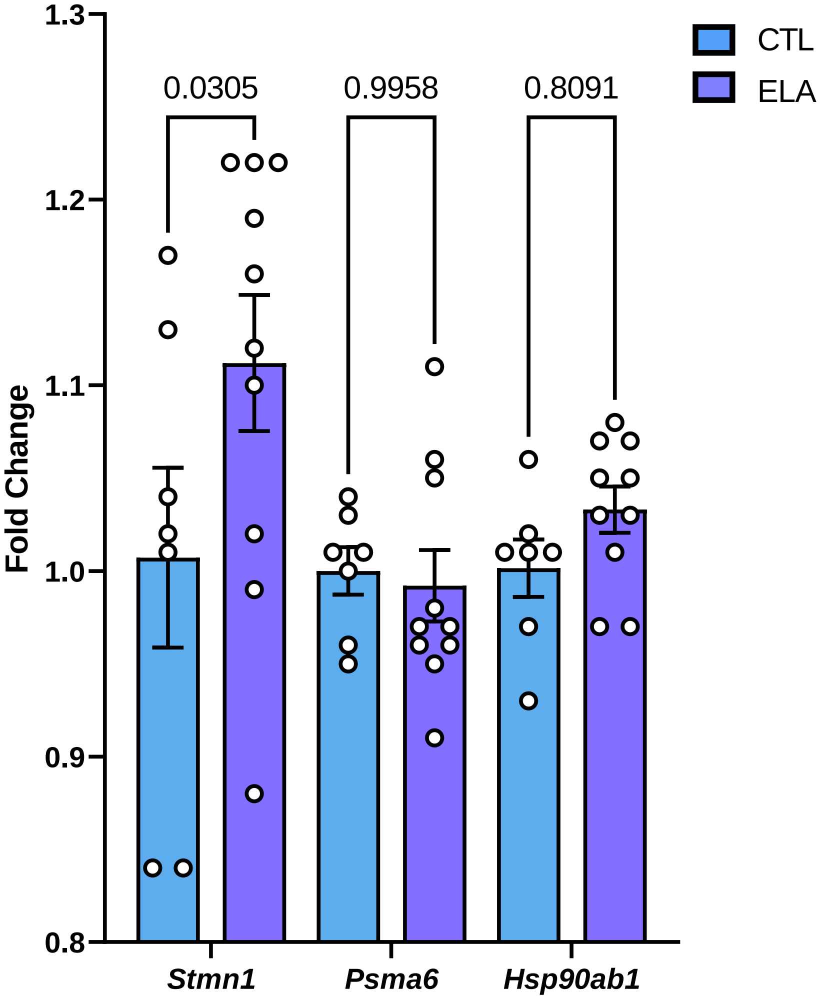

Supplementary Figure S4. Enduring upregulation of neuronal structural gene *Stmn1* in adult bulk PVN. qPCR was performed on a subset of genes that were upregulated following ELA in our single-cell analyses. We employed whole PVN punches from adult male mice. The neuronal structure-related gene *Stmn1* was enduringly upregulated in whole PVN of adult ELA animals, whereas genes related to immediate response to stress (*Psma6* and *Hsp90ab1*) were not significantly changed. Fold change was calculated using ΔΔCT and analysis was performed using 2way ANOVA with gene as a repeated factor ($F_{(1,15)}$=2.24, p=0.15), a main effect of experience ($F_{(2,30)}$=3.9, p=0.03), and gene x experience interaction ($F_{(2,30)}$=3.06, p=0.06). P values represent planned post-hoc analysis for the effect of ELA within each gene. Dots represent individual mice; bars represent mean ±SEM

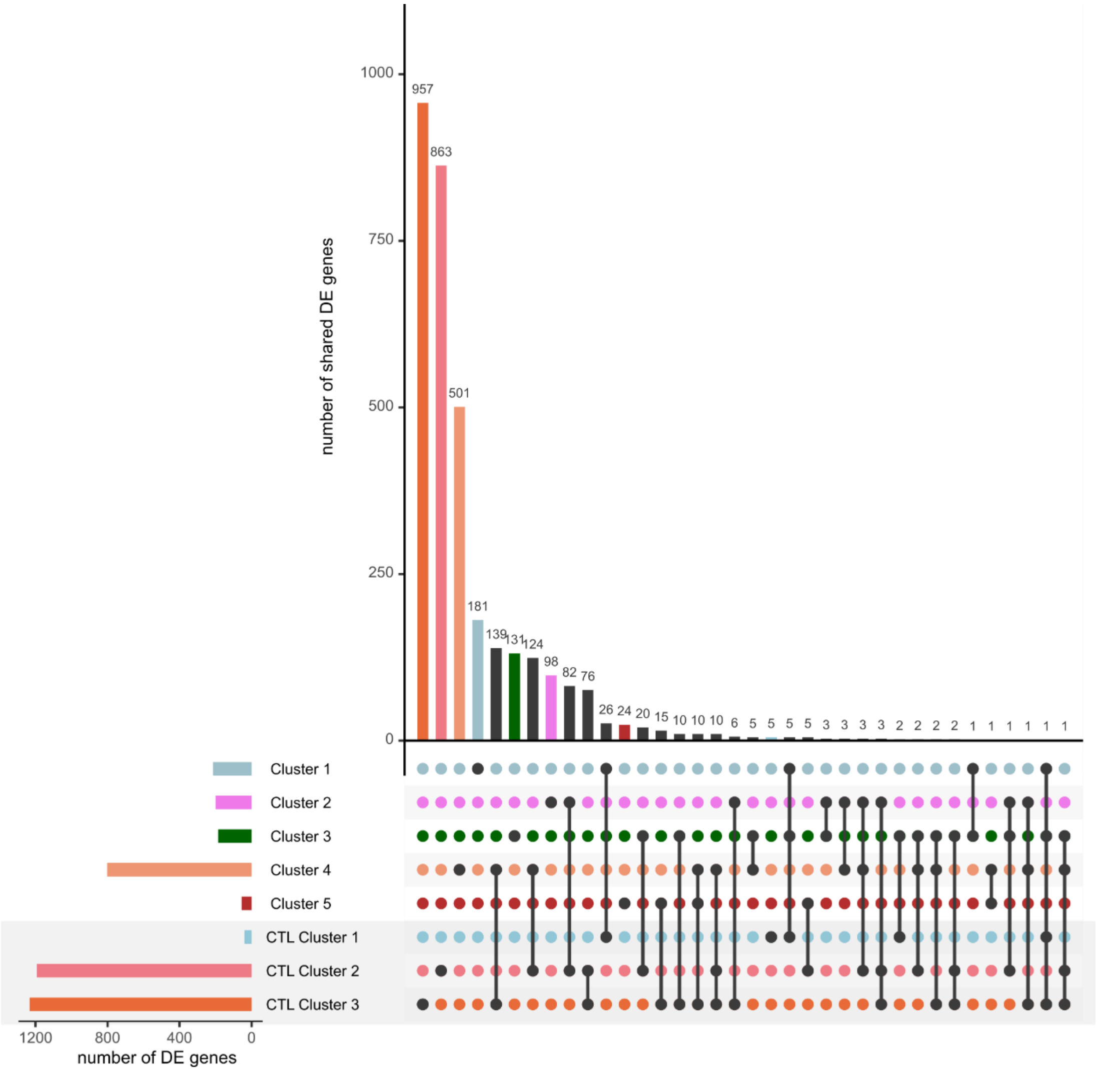

Supplementary Figure S5. Representation of the number of shared differentially expressed genes per cluster. Most clusters including CTL and ELA cells had distinct DE genes. To exclude the possibility that ELA might modulate the expression state or clustering of the population of PVN-CRH cells in the developing mouse, we performed the same cluster analysis on CTL cells only (in grey box). Given that the cell number was now lower, the results - three clusters that encompassed most genes from the above clusters - supported the validity of this clustering approach: CTL cluster 1 contained genes from clusters 1 and 3, CTL cluster 2 contained genes also expressed in cluster 2. The third CTL cluster contained more unique genes but also included genes from clusters 4 and 5. In sum, segregation of PVN-CRH cells into several biologically distinct clusters was not driven by ELA-induced changes to transcription.

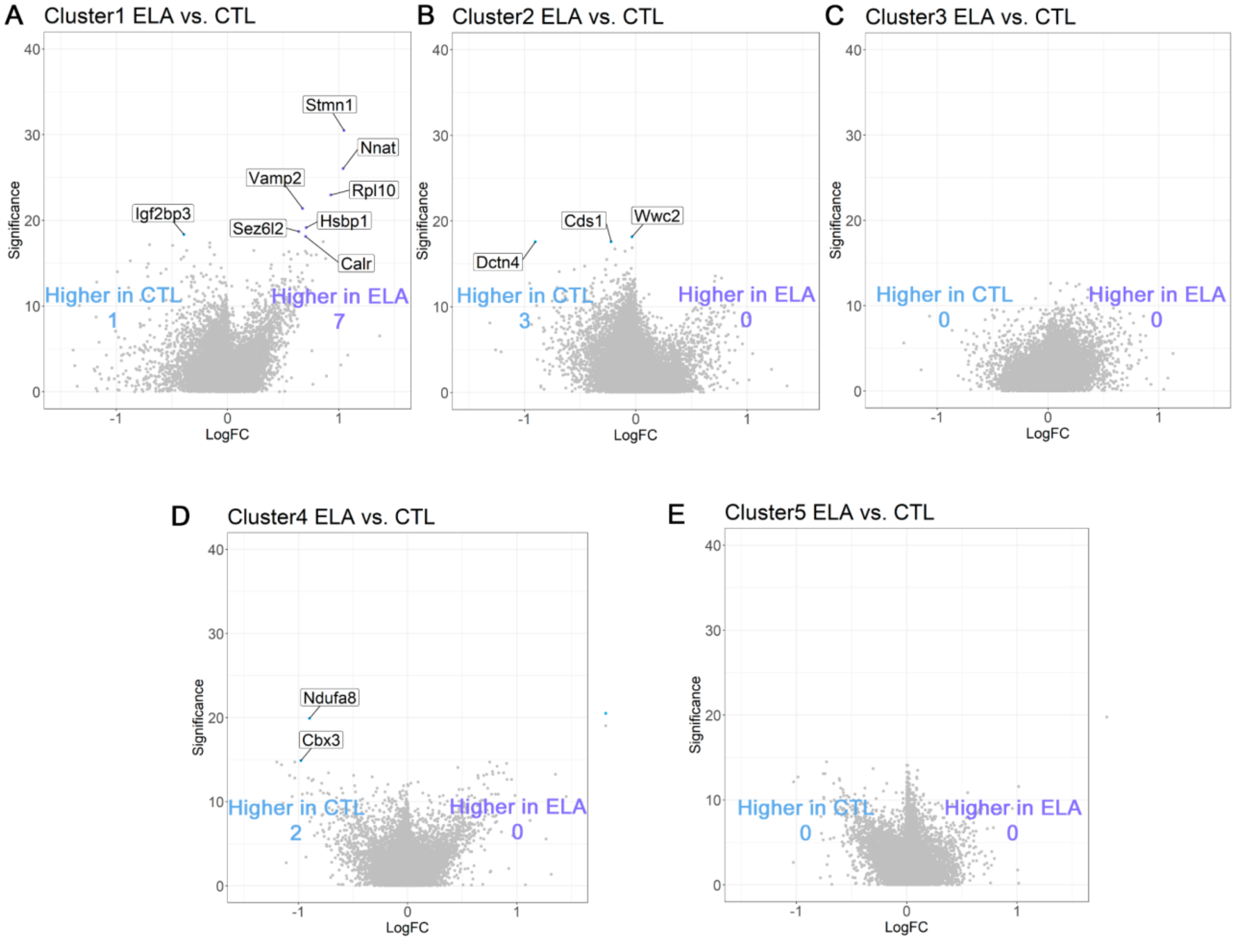

Supplementary Figure S6. Differential expression analysis in each Seurat cluster between the ELA and CTL cells. Volcano plots representing differential expression between the ELA and CTL cells. (A) In cluster 1, there were 7 genes enriched in ELA cells: *Stmn1, Nnat* (Neuronatin)*, Rpl10* (Ribosomal Protein L10)*, Vamp2, Hsbp1* (Heat Shock Factor Binding Protein 1)*, Sez6l2* (Seizure Related 6 Homolog Like 2)*,* and *Calr* (Calreticulin) and one gene enriched in CTL: *Igf2bp3* (Insulin Like Growth Factor 2 MRNA Binding Protein 3. Three of these genes were also enriched in the overall population of ELA vs CTL cells, (Figure 1B), four genes (*Nnat, Hsbp1, Sez6l2*, and *Calr*) were specifically enriched in cluster 1 following ELA (B) In cluster 2, there were 0 differentially expressed genes enriched in ELA cells and 3 in the CTL cells (*Wwc2* (WW And C2 Domain Containing 2) (C) No differentially expressed genes were observed in cluster 3. (D) There were 0 differentially expressed genes enriched in ELA cells and 2 in the CTL cells in cluster 4 (*Ndufa8* (NADH:Ubiquinone Oxidoreductase Subunit A8), which were not observed in the total-population analyses. (E) No differentially expressed genes were present in cluster 5. All enriched genes were assessed for false discovery rates (FDR) <0.1.

Supplementary References

1. Gilles EE, Schultz L, Baram TZ (1996): Abnormal corticosterone regulation in an immature rat model of continuous chronic stress. *Pediatric Neurology* 15: 114–119.

2. Molet J, Maras PM, Avishai-Eliner S, Baram TZ (2014): Naturalistic rodent models of chronic early-life stress. *Developmental Psychobiology* 56: 1675–1688.

3. Rice CJ, Sandman CA, Lenjavi MR, Baram TZ (2008): A novel mouse model for acute and long-lasting consequences of early life stress. *Endocrinology* 149: 4892–4900.

4. Molet J, Heins K, Zhuo X, Mei YT, Regev L, Baram TZ, Stern H (2016): Fragmentation and high entropy of neonatal experience predict adolescent emotional outcome. *Translational Psychiatry* 6: e702–e702.

5. Brunson KL, Kramár E, Lin B, Chen Y, Colgin LL, Yanagihara TK, *et al.* (2005): Mechanisms of late-onset cognitive decline after early-life stress. *The Journal of neuroscience : the official journal of the Society for Neuroscience* 25: 9328–38.

6. Avishai-Eliner S, Gilles EE, Eghbal-Ahmadi M, Bar-El Y, Baram TZ (2001): Altered Regulation of Gene and Protein Expression of Hypothalamic-Pituitary-Adrenal Axis Components in an Immature Rat Model of Chronic Stress. *Journal of Neuroendocrinology* 13: 799–807.

7. Chen Y, Molet J, Gunn BG, Ressler K, Baram TZ (2015): Diversity of Reporter Expression Patterns in Transgenic Mouse Lines Targeting Corticotropin-Releasing Hormone-Expressing Neurons. *Endocrinology* 156: 4769–80.

8. Gunn BG, Sanchez GA, Lynch G, Baram TZ, Chen Y (2019): Hyper-diversity of CRH interneurons in mouse hippocampus. *Brain Structure and Function* 224: 583–598.

9. Daviu N, Füzesi T, Rosenegger DG, Rasiah NP, Sterley TL, Peringod G, Bains JS (2020): Paraventricular nucleus CRH neurons encode stress controllability and regulate defensive behavior selection. *Nature Neuroscience* 23: 398–410.

10. Daviu N, Fuzesi T, Rosenegger D, Peringod G, Simone K, Bains J (2020): Visual-looming Shadow Task with in-vivo Calcium Activity Monitoring to Assess Defensive Behaviors in Mice. *BIO-PROTOCOL* 10. https://doi.org/10.21769/bioprotoc.3826

11. Hokenson R, Oijala M, Short A, Bolton J, Chen Y, Molet J, *et al.* (2020): Multiple Simultaneous Acute Stresses in Mice: Single or Repeated Induction. *BIO-PROTOCOL* 10. https://doi.org/10.21769/BioProtoc.3699

12. Chen Y, Molet J, Lauterborn JC, Trieu BH, Bolton JL, Patterson KP, *et al.* (2016): Converging, Synergistic Actions of Multiple Stress Hormones Mediate Enduring Memory Impairments after Acute Simultaneous Stresses. *The Journal of Neuroscience* 36: 11295–11307.

13. Maras PM, Molet J, Chen Y, Rice C, Ji SG, Solodkin A, Baram TZ (2014): Preferential loss of dorsal-hippocampus synapses underlies memory impairments provoked by short, multimodal stress. *Molecular Psychiatry* 19: 811–822.

14. Libovner Y, Fariborzi M, Tabba D, Ozgur A, Jafar T, Lur G (2020): Repeated Exposure to Multiple Concurrent Stresses Induce Circuit Specific Loss of Inputs to the Posterior Parietal Cortex. *Journal of Neuroscience* 40: 1849–1861.

15. Hokenson RE, Short AK, Chen Y, Pham AL, Adams ET, Bolton JL, *et al.* (2021): Unexpected role of physiological estrogen in acute stress-induced memory deficits. *The Journal of Neuroscience* 41: 648–662.
